# Expression patterns and prognostic value of Bag-1 and Bcl-2 in breast cancer

**DOI:** 10.1186/bcr1998

**Published:** 2008-04-23

**Authors:** Yasmine Nadler, Robert L Camp, Jennifer M Giltnane, Christopher Moeder, David L Rimm, Harriet M Kluger, Yuval Kluger

**Affiliations:** 1 Department of Medicine, Yale University School of Medicine, New Haven, CT 06520, USA; 2 Department of Pathology, Yale University School of Medicine, New Haven, CT 06520, USA; 3 Department of Cell Biology, New York University School of Medicine, 540 First Avenue, 3rd Floor, New York, NY 10016, USA

## Abstract

**Introduction:**

Bcl-2 antanogene-1 (Bag-1) binds the anti-apoptotic mediator Bcl-2, and enhances its activity. Bcl-2 and Bag-1 are associated with chemotherapy resistance in cancer cells. Drugs that target Bcl-2 are currently in clinical development. The purpose of the present study was to examine expression patterns of Bag-1 in a large cohort of breast tumors and to assess the association with Bcl-2, estrogen receptor, progesterone receptor and Her2/neu, and other clinical/pathological variables.

**Methods:**

Tissue microarrays containing primary specimens from 638 patients with 10-year follow-up were employed, and the expression of Bag-1, Bcl-2, estrogen receptor, progesterone receptor and Her2/neu was assessed using our automated quantitative analysis method. We used cytokeratin to define pixels as breast cancer (tumor mask) within the array spot, and we measured biomarker expression within the mask using Cy5 conjugated antibodies.

**Results:**

High Bcl-2 expression was associated with improved survival in the entire cohort and in the node-positive subset (*P *= 0.008 and *P = *0.002, respectively). High Bag-1 expression was associated with improved survival in the node-positive subset (*P = *0.006). On multivariable analysis, neither Bcl-2 nor Bag-1 retained their independence as prognostic markers. Strong associations were found between Bag-1, Bcl-2, estrogen receptor and progesterone receptor.

**Conclusion:**

Bag-1 and Bcl-2 expression in breast tumors is associated with improved outcome and steroid receptor positivity. Evaluation of Bcl-2 and Bag-1 expression in breast cancer may identify a subset of patients with a favorable prognosis, who might not benefit from chemotherapy or who might benefit from Bcl-2 targeting agents in addition to antihormonal therapy.

## Introduction

Breast cancer is the most common malignancy among women, with a projected incidence of 178,480 new diagnoses in the United States in 2007 [1]. Over 40,000 women are expected to die from metastatic disease in 2007 [1]; adjuvant systemic therapy is therefore given for early-stage disease to decrease the risk of death from breast cancer.

A number of factors are used to assess the risk of developing metastatic disease and death, including lymph node involvement, tumor size, nuclear and histologic grade, age, hormone receptor expression and Her2/neu status. Lymph node involvement is the most reliable predictor of metastatic relapse, yet within the lymph node-positive subset and the lymph node-negative subset of patients there is variability in prognosis, and we have no reliable means of determining which patients will survive without adjuvant systemic therapy. For example, it is well established that adjuvant chemotherapy decreases the risk of recurrence in node-positive patients [2], yet older studies showed that there is a subset of node-positive breast cancer patients, particularly those with estrogen receptor (ER)-positive tumors, who survive with tamoxifen alone [3]. There is therefore great need to identify new prognostic markers that will assist in patient selection for adjuvant therapies. Moreover, these markers can assist in selection of biospecific therapies once drugs that target these markers become available.

A number of prior studies have assessed the prognostic value of the anti-apoptotic mediator Bcl-2 in breast cancer [4-24]. Bcl-2 blocks apoptosis via the mitochondrial pathway by inhibiting the release of cytochrome C from the mitochondria, thus preventing the cascade of events that results in compromise of the mitochondrial outer membrane potential, which in turn leads to caspase-9 activation and subsequent apoptosis [25].

Bcl-2 has been shown to inhibit chemotherapy-induced apoptosis, and chemotherapy resistance has been reversed in cancer cells treated with Bcl-2-targeting therapy [26]. Although Bcl-2 is an anti-apoptotic protein, high Bcl-2 expression has been observed in ER-positive breast cancers [4,8,13,14,18,20,23,27-38], as well as in progesterone receptor (PR)-positive breast cancers [4,13,14,27-31,33-39], and has been associated with improved survival in breast cancer [4-24,40]. The largest of these studies – conducted by Callagy and colleagues – included 930 cases, and showed that positive Bcl-2 expression was a strong predictor of improved survival, independent of the Nottingham prognostic index [6]. High Bcl-2 expression has been associated with improved prognosis even among patients at very high risk for distant relapse, with over 10 involved lymph nodes [41].

Bcl-2 antanogene-1 (Bag-1) is a protein that has multiple cellular functions. Bag-1 binds to Bcl-2 and enhances its anti-apoptotic activity [42,43]. Bag-1 also has anti-apoptotic effects that are independent of Bcl-2; it binds to multiple receptor tyrosine kinases and enhances their ability to inhibit apoptosis [44], and it interacts with the heat shock proteins HSC70 and HSP70 [45]. Bag-1 modulates the function of the ER, and enhances estrogen-dependent transcription [46]. By binding to its partners and regulating their function, Bag-1 therefore modulates pathways necessary for transcription and cell growth and survival, as reviewed by Townsend and colleagues [47].

The Bag-1 gene has four protein isoforms, three major isoforms (p50, p46 and p33 – Bag-1L, Bag-1M and Bag-1S, respectively) and one minor isoform (p29) [48,49]. The isoforms arise from a single mRNA by alternative translation initiation [50,51]. The distinct isoforms are associated with different subcellular locations and have variable functions [49,52]. Bag-1L is localized to the nucleus, whereas Bag-1M and Bag-1S are generally found in the cytoplasm. In some cell types and in conditions of stress, however, Bag-1M may also be localized to the nucleus [49,52].

Several preclinical studies have demonstrated the importance of Bag-1 in breast cancer [19,42,53-60]. In breast cancer cell lines, Bag-1 prevents cells from undergoing apoptosis and protects cells from other forms of stress, including radiation, chemotherapy and hypoxia [61]. ZR-75-1 breast cancer cells stably transfected with Bag-1 have increased survival in culture, and form larger tumors than nontransfected cells when injected into mammary fat pads of mice [62]. ZR-75-1 cells stably expressing mutated forms of Bag-1 display retarded growth *in vivo *and *in vitro *[62], suggesting that targeting Bag-1 might be a useful strategy for treating breast cancer.

A number of relatively small cohort studies have assessed expression of Bag-1 in breast cancer [19,42,52-60,63], with divergent results. For example, Tang and colleagues studied 140 breast tumors and found an association between high nuclear Bag-1 expression and decreased survival [58], Townsend and colleagues found no significant association between nuclear or cytoplasmic Bag-1 and survival in 160 patients [59], and Turner and colleagues found a strong association between high cytoplasmic Bag-1 and improved survival [60].

Similarly, reported associations between Bag-1 and Bcl-2, ER and PR are variable; some researchers have reported positive correlations between Bag-1 and Bcl-2 expression [56,57,60], while other workers have made the opposite observation [42,52]. Some studies have shown no association between Bag-1 expression and ER or PR expression [58-60], whereas other studies have shown that Bag-1 and ER do tend to coexpress [46,52,57], as do Bag-1 and PR [46,57]. These inconsistencies could be due to relatively small cohorts, technical variability in staining from specimen to specimen, and lack of quantitative measures for immunohistochemistry.

To address these issues, we assessed expression of Bag-1 on a large cohort of primary breast cancers using tissue microarrays, employing a new method of automated, quantitative analysis. This method has been shown to be more accurate than pathologist-based scoring of 3,3'-Diaminobenzidine Tetrahydrochloride stain (DAB) stain [64], and produces quantitative measures that are directly proportional to the concentration of the measured biomarker [65,66]. Since the ER, PR, Her2/neu and Bcl-2 have prognostic importance in breast cancer and are also targets of drugs that are in clinical use, we also assessed the association between Bag-1 expression and these markers, with the goal of characterizing subsets of patients based on expression of these biomarkers. We found that Bag-1 and Bcl-2 tend to be coexpressed, and expression is correlated with ER and PR expression. Both Bag-1 and Bcl-2 were associated with improved survival among node-positive breast cancer patients, particularly those with hormone receptor-positive tumors.

## Materials and methods

### Cell lines and western blots

The MDA-MB-468, MCF-7, T47D, BT-474 and SKBR3 (human breast cancer) cell lines were purchased from ATCC (Manassas, VA, USA). Western blotting of protein extracts was performed using standard methods. Bag-1 and Bcl-2 expression were detected by overnight incubation with mouse anti-Bag-1 IgG (Chemicon, Temecula, CA, USA) at 1:400 and with mouse anti-Bcl-2 IgG (Dako Corp., Carpinteria, CA, USA) at 1:6,000. Protein loading was assessed using rabbit anti-β-actin (Sigma-Aldrich, St Louis, MO, USA) at 1:5,000.

### Tissue microarray construction

The breast cancer tissue microarrays were constructed as previously described [67]. A total of 319 node-negative and 319 node-positive breast cancer cores, each measuring 0.6 mm in diameter, were spaced 0.8 mm apart on two glass slides. The cohort was constructed from paraffin-embedded, formalin-fixed tissue blocks obtained from the Yale University Department of Pathology Archives. Specimens and clinical information were collected under the guidelines and approval of a Yale University Institutional Review Board.

By standard immunohistochemistry, ER staining was positive in 52%, PR in 46% and HER2/neu in 14% of specimens. Nuclear grade 3 (on a 1 to 3 scale) was seen in 28% of the specimens, and 59% were larger than 2 cm. The histological subtypes included 72% invasive ductal carcinoma and 1% lobular carcinoma, and 14% had mixed or other histology. The specimens were resected between 1962 and 1983, with a follow-up range between 4 months and 53 years, and a mean follow-up time of 12.6 years. Patient age at diagnosis ranged from 24 to 88 years (mean age, 58 years).

A complete treatment history was not available for the entire cohort. Most patients were treated with local irradiation. None of the node-negative patients were given adjuvant systemic therapy. A minority of the node-positive patients (approximately 15%) received chemotherapy, and approximately 5.6% of patients received tamoxifen (postmenopausal patients with ER-positive tumors, treated after 1978). The time between tumor resection and tissue fixation was not available.

A pathologist reviewed slides from all of the blocks to select representative areas of invasive tumor to be cored. The cores were placed on the tissue microarray using a Tissue Microarrayer (Beecher Instruments, Silver Spring, MD, USA). The tissue microarrays were then cut into 0.5 μm sections and were placed on glass slides using an adhesive tape-transfer system (Instrumedics, Inc., Hackensack, NJ, USA) with UV cross-linking.

### Immunohistochemistry

Staining was performed for automated analysis of breast cancer specimens as previously described [68]. Briefly, slides were deparaffinized in xylene, and were transferred through two changes of 100% ethanol. For antigen retrieval, the slides were pressure-cooked in 6.5 mM sodium citrate (pH 6.0). Endogenous peroxidase activity was blocked in a mixture of methanol and 2.5% hydrogen peroxide for 30 minutes. To reduce nonspecific background staining, slides were incubated for 30 minutes in 0.3% bovine serum albumin/1 × Tris-buffered saline.

Slides were then incubated at 4°C overnight with the following primary antibodies: mouse monoclonal anti-Bcl-2 (clone 124; Dako) at 1:40; mouse monoclonal anti-Bag-1 (MAB4611; Chemicon) at 1:150; mouse monoclonal anti-ER (Dako) at 1:50; mouse monoclonal anti-PR at 1:50 (Dako); and rabbit anti-Her2/neu at 1:8,000 (Dako). All antibodies were diluted in Tris-buffered saline containing 0.3% bovine serum albumin. Goat anti-mouse (or anti-rabbit for Her2/neu) horseradish peroxidase-decorated polymer backbone (Envision; Dako) was used as a secondary reagent, and Cy5-tyramide (Perkin Elmer Life Science, Waltham, MA, USA) was used to visualize the target.

To create a tumor mask, primary slides were simultaneously incubated with rabbit anti-human cytokeratin antibodies diluted at 1:200. For Bcl-2, Bag-1, ER and PR, rabbit anti-cytokeratin was used. Mouse anti-cytokeratin was used for Her2/neu. The anti-cytokeratin antibodies were visualized with secondary Alexa 488-conjugated goat anti-rabbit or goat anti-mouse antibodies (Molecular Probes, Inc., Eugene, OR, USA). Coverslips were mounted with ProLong Gold antifade reagent with 4',6-diamidino-2-phenylindole (Invitrogen Corp, Grand Island, NY, USA).

### Automated image acquisition

Images were acquired using automated quantitative analysis (AQUA), as described previously [67]. Briefly, areas of tumor were distinguished from stroma by creating a mask with the cytokeratin signal tagged with Alexa 488. Coalescence of cytokeratin at the cell surface was used to identify the membrane/cytoplasm compartment within the tumor mask, while 4',6-diamidino-2-phenylindole (DAPI) was used to identify the nuclear compartment within the tumor mask. The target markers, Bag-1, Bcl-2, ER, PR or Her2/neu, were visualized with Cy5 (red). Multiple monochromatic, high-resolution (1,024 × 1,024 pixels, 0.5 μm) grayscale images were obtained for each histospot, using the 10 × objective of an Olympus AX-51 epifluorescence microscope (Olympus, Melville, NY, USA) with an automated microscope stage and digital image-acquisition driven by custom program and macro-based interfaces with IPLabs software (Scanalytics Inc., Fairfax, VA, USA).

### Algorithmic image analysis

Images were analyzed using algorithms that have been previously extensively described [68]. Two images (one in-focus and one out-of-focus) were taken of the compartment-specific tags and the target marker. A percentage of the out-of-focus image was subtracted from the in-focus image for each pixel, representing the signal-to-noise ratio of the image. An algorithm called the Rapid Exponential Subtraction Algorithm was used to subtract the out-of-focus information in a uniform fashion for the entire microarray. Subsequently, a second algorithm called the Pixel Locale Assignment for Compartmentalization of Expression algorithm was used to assign each pixel in the image to a specific subcellular compartment, and the signal in each location was calculated. The data were expressed as the average signal intensity per unit of compartment area on a scale of 0 to 255, and were expressed as target signal intensity relative to the compartment area.

### Statistical analysis

The JMP5 software package (SAS Institute Inc., Cary, NC, USA) was used for data analysis. Continuous AQUA scores of target expression were dichotomized by the median score, and associations with clinical and pathological parameters were completed using unpaired *t *tests. The prognostic significance of the parameters was assessed using the Cox proportional hazards model with breast cancer-specific survival as an endpoint. Survival curves were generated using the Kaplan–Meier method, with significance evaluated using the Mantel–Cox long-rank test. Associations between continuous AQUA scores for the different markers were assessed by the Spearman's rho test.

**Figure 1:**
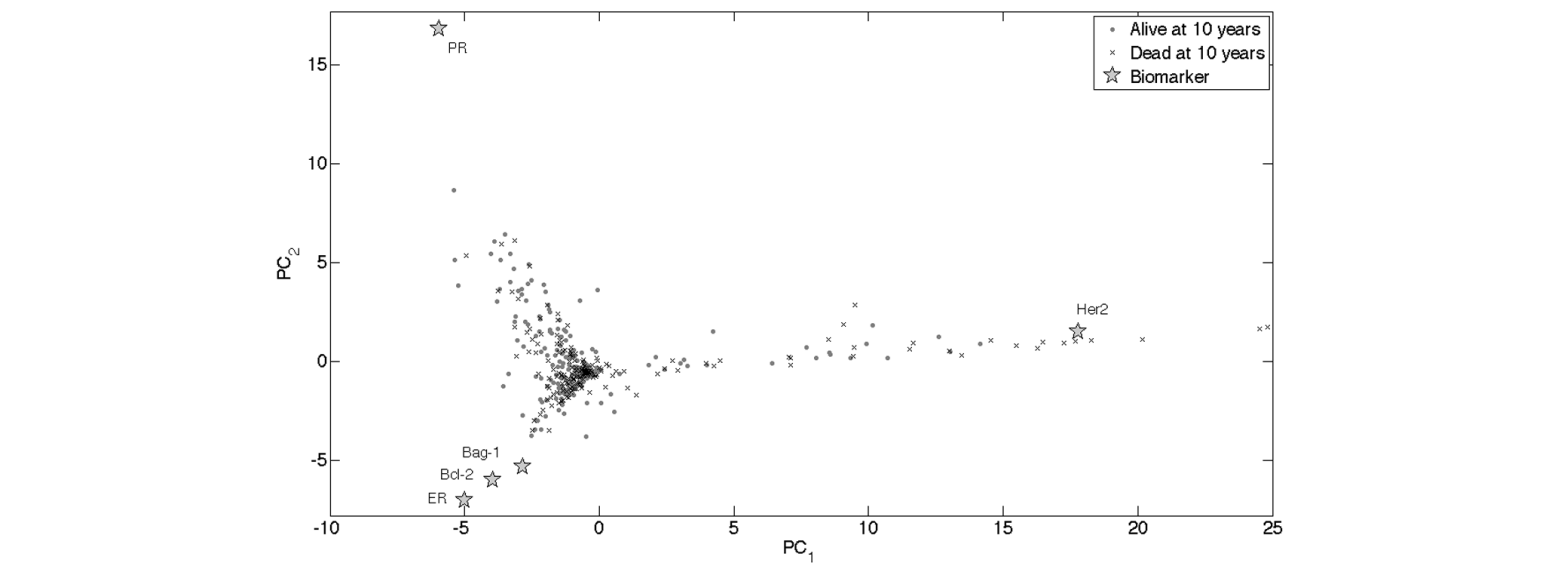
**Principal component analysis and biplot**. Projection of five-dimensional patient biomarker profiles with no missing data (439 instances) and the 439-dimensional biomarkers profiles onto the two leading principal components of a matrix consisting of expression profiles of Her2/neu, estrogen receptor (ER), progesterone receptor (PR), Bcl-2 and Bcl-2 antanogene-1 (Bag-1) present in all 439 samples. Each patient is represented by a distinct symbol (•, alive at 10 years; ×, dead at 10 years). The accumulated variation captured by the first and second principal components is 92% of the total variation. Overlaying a two-dimensional scatter plot representing the projection of the biomarkers () onto the first and second principal components on top of the two-dimensional patient scatter plot representing the projection of their five-dimensional biomarker profiles onto the two leading principal components forms a biplot. The biplot can be used to read the approximated transformed expression levels.

To generate the principal component analysis biplot (presented in Figure 1), we tabulated our dataset in a matrix consisting of 439 patients (rows) and five biomarkers (columns). We only included the 439 patients (out of 638 patients) who had values for all five biomarkers. The first step in this analysis involves data preprocessing obtained by first normalizing each marker 439-dimensional profile by its median value, followed by a two-way data-centering procedure of this normalized data matrix. The centering procedure involves transforming each entry of this matrix by subtraction of the row and column means of this entry and addition of the overall matrix mean, leading to a transformed matrix having all row sums and all column sums equal to zero. Reduction in the number of variables is useful for visualizing how the patient population is distributed in the five-dimensional variable space. Principal component analysis is an unsupervised dimension reduction method that generates a new set of decorrelated variables (principal components) as linear combinations of the original variables (biomarkers). The majority of the variation associated with the Her2/neu, ER, PR, Bcl-2 and Bag-1 variables can be captured by the most dominant principal components. An additional advantage of expressing the data in terms of the leading principal components is their robustness to noise.

The projections of the samples onto the leading principal components were computed by applying the singular value decomposition to the data matrix (after preprocessing as described above and in previous works [69]). We used principal component biplots to display the biomarkers (columns of the data matrix) and the patients (rows of the data matrix) simultaneously as points in a two-dimensional space [70]. The biplot provides an optimal approximation of the data matrix by such a two-dimensional structure, in that it displays the singular value decomposition – which gives the rank-two approximation to the data matrix having the smallest mean-squared error. The expression of a given biomarker in a given patient sample is approximated by the projection of the biomarker vector onto the direction of the patient sample vector, multiplied by the length of the patient sample vector. In the present rank-two approximation, therefore, for a given biomarker and for patient vectors of a given length, the biomarker is expressed at a higher (or lower) level in patients whose vector points in nearly the same (or opposite) direction as the biomarker. A biomarker is not overexpressed or underexpressed for patients whose vector is orthogonal to the biomarker vector, and is underexpressed for patients whose vector form an obtuse angle with the biomarker vector.

## Results

### Western blots for Bag-1 and Bcl-2

**Figure 2:**
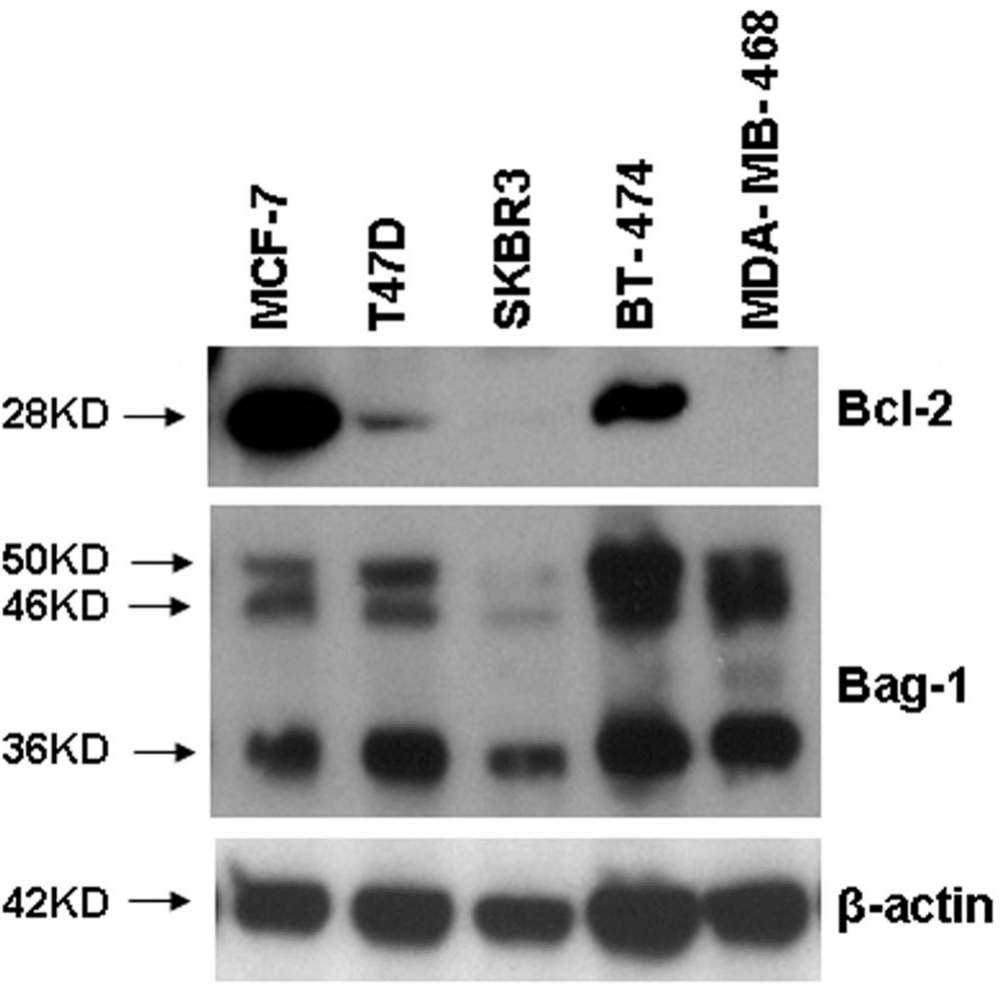
**Expression of Bcl-2 antanogene-1 isoforms and Bcl-2**. Expression of Bcl-2 antanogene-1 (Bag-1) isoforms and Bcl-2, using β-actin as a loading control in a panel of breast cancer cell lines.

Western blot analysis for Bag-1 showed bands at 36 kDa, 46 kDa and 50 kDa (Figure 2). These represent the three isoforms reported in the literature [49], and all three isoforms are recognized by the antibody. The strongest expression of Bag-1 was observed in BT-474, consistent with findings by other researchers [42]. For Bcl-2, a single 28 kDa band was seen; expression was high in the MCF-7 cell line and low in the SKBR3 cell line, as reported in the literature [26,42,52].

### Immunohistochemistry

**Figure 3:**
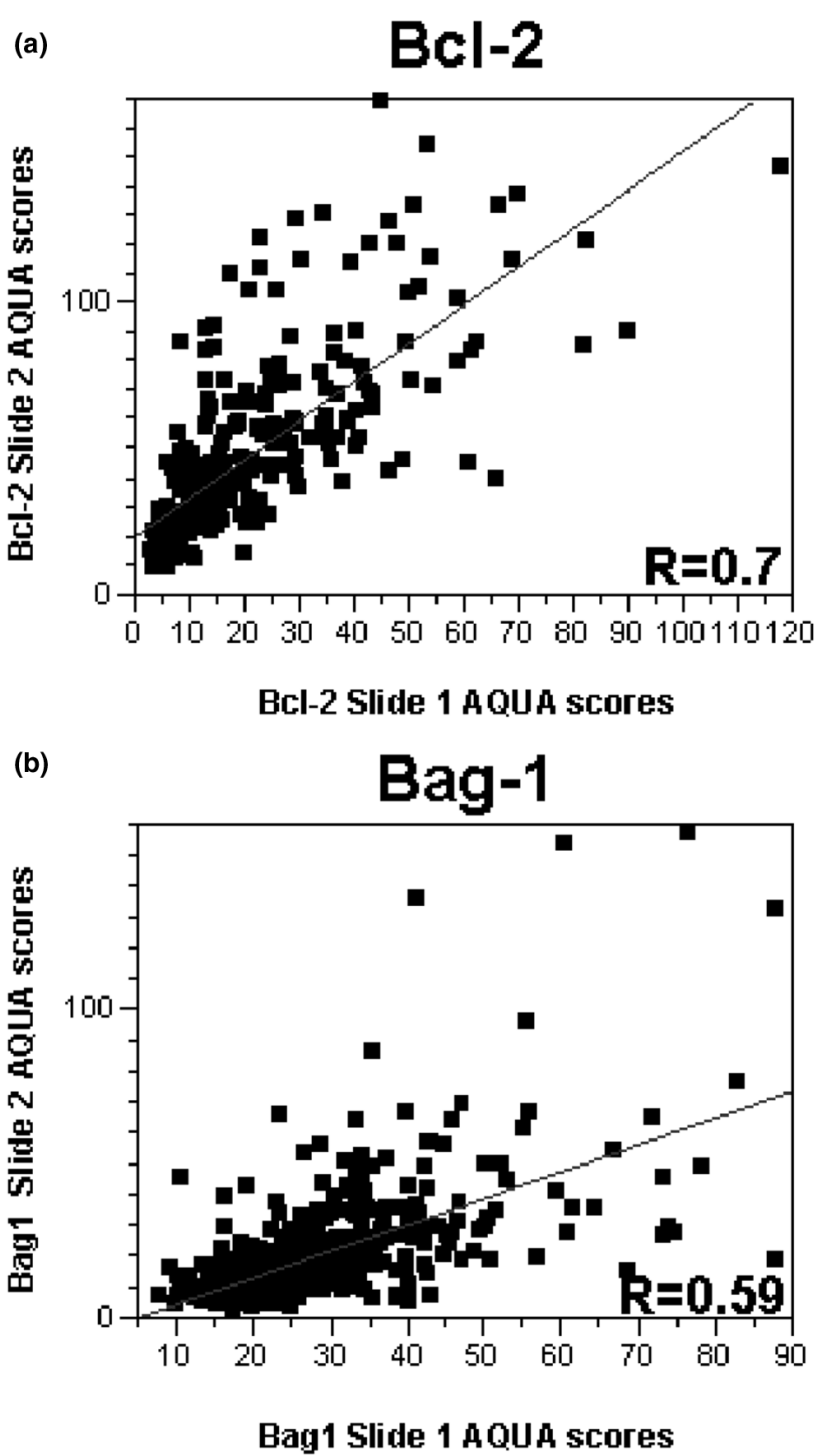
**Regression plot for scores from breast cancer arrays stained for Bcl-2 and Bcl-2 antanogene-1**. Regression plot for scores from the two breast cancer arrays stained for **(a) **Bcl-2 and **(b) **Bcl-2 antanogene-1 (Bag-1). AQUA, automated quantitative analysis.

**Figure 4:**
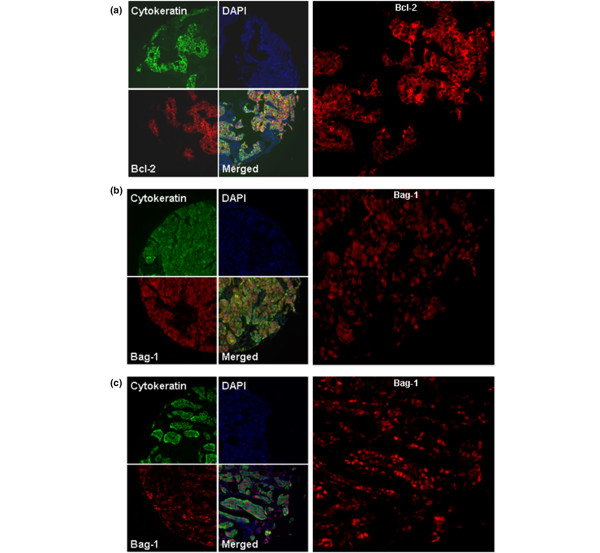
**Immunoflourescent staining of Bcl-2 and Bcl-2 antanogene-1 in breast tumor tissue**. **(a) **Cytoplasmic Bcl-2, **(b) **cytoplasmic and nuclear Bcl-2 antanogene-1 (Bag-1) and **(c) **nuclear Bag-1 staining in a breast cancer histospot – using cytokeratin to the define tumor mask, using 4',6-diamidino-2-phenylindole to define the nuclear compartment, and using Cy5 for identifying the target (Bcl-2 and Bag-1).

To account for intratumor heterogeneity, two separate sets of slides – each containing a core from a different area of the tumor for each patient – were used to evaluate the expression of each marker. Bcl-2 and Her2/neu did not have significant amounts of nuclear staining, and only the membranous/cytoplasmic compartments were analyzed, and *vice versa*, for ER and PR staining. Bag-1 staining was either nuclear or cytoplasmic, and many specimens had both nuclear and cytoplasmic staining. We performed log-regression analyses to assess the correlation between the two slides for Bag-1 and Bcl-2, as demonstrated in Figure 3. The matching spots on each array were highly correlated for all four markers (*R *= 0.6 for Bag-1, *R *= 0.7 for Bcl-2, *R *= 0.74 for the ER, *R *= 0.79 for the PR and *R *= 0.92 for Her2/neu). AQUA scores ranged from 8.3 to 146.02 for the total Bag-1 score (median, 24.07), from 8.37 to 259.61 for Bcl-2 (median, 32.04), from 2.34 to 94.097 for the PR (median, 6.58), from 1.085 to 61.164 for Her2/neu (median, 2.03), and from 2.29 to 105.39 for the ER (median, 13.46). Examples of Bag-1 and Bcl-2 staining are shown in Figure 4.

For each of the markers, the AQUA scores from both sets of slides were combined to give a single dataset. Tumor spots were deemed uninterpretable if they had insufficient tumor cells, loss of tissue in the spot or an abundance of necrotic tissue. For patients who had two interpretable histospots, a composite score was formed by taking the average of the two scores. For patients with only one interpretable core, the single score was used. The combined dataset for Bag-1 had scores for 574 patients. We obtained scores for Bcl-2, Her2/Neu, ER and PR for 528 patients, 608 patients, 601 patients and 595 patients, respectively.

Given that the nodal status often determines the standard clinical approach to patients, we assessed the prognostic value of Bag-1 and Bcl-2 in the entire cohort, as well as within the node-positive and node-negative subsets of patients. Using the Cox univariate survival analysis of raw AQUA scores, we found that Bag-1 expression (nuclear, cytoplasmic and total) was associated with breast cancer-specific survival in the node-positive subset only (*P = *0.006 for the total Bag-1 score), whereas Bcl-2 expression was associated with survival in the entire cohort and in the node-positive subset (*P *= 0.008 and *P *= 0.002, respectively). The association with survival for Bag-1 and Bcl-2 within the node-positive subset (but not the node-negative subset) might be due to the larger number of events (deaths) within the node-positive subset.

Table 1 presents the associations between continuous scores of Bag-1, Bcl-2, ER, PR and Her2/neu and survival in the entire cohort and in the node-negative and node-positive subsets. There were no remarkable differences between nuclear Bag-1 scores, cytoplasmic Bag-1 scores and total Bag-1 scores as predictors of survival; the remainder of these analyses will therefore focus on the total Bag-1 scores.

**Table 1:** Association between Bcl-2 antanogene-1 (Bag-1), Bcl-2, estrogen receptor, progesterone receptor and Her2/neu and breast cancer-specific survival by Cox univariate analysis with 10-year follow-up

Patient subset	Bag-1 (general)	Bag-1 (nuclear)	Bcl-2	Estrogen receptor	Progesterone receptor	Her2/neu
Node positive	*P *= **0.006**, n = 259, HR = 0.98 (0.97 to 0.995)	*P *= **0.008**, n = 259, HR = 0.987 (0.978 to 0.997)	*P *= **0.002**, n = 242, HR = 0.99 (0.98 to 0.997)	*P *= **0.0033**, n = 270, HR = 0.988 (0.98 to 0.996)	*P *= **0.0012**, n = 270, HR = 0.97 (0.96 to 0.99)	*P *= **0.0091**, n = 272, HR = 1.02 (1.006 to 1.036)
Node-negative	*P *= 0.67, n = 259, HR = 0.997 (0.98 to 1.01)	*P *= 0.47, n = 259, HR = 0.995 (0.98 to 1.007)	*P *= 0.59, n = 240, HR = 0.998 (0.99 to 1.004)	*P *= 0.57, n = 273, HR = 0.997 (0.98 to 1.008)	*P *= 0.25, n = 266, HR = 0.99 (0.97 to 1.006)	*P *= 0.22, n = 276, HR = 1.02 (0.98 to 1.05)
All patients	*P *= 0.08, n = 518, HR = 0.99 (0.98 to 1.001)	*P *= 0.06, n = 517, HR = 0.99 (0.985 to 1)	*P *= **0.008**, n = 482, HR = 0.99 (0.989 to 0.999)	*P *= **0.01**, n = 543, HR = 0.99 (0.984 to 0.998)	*P *= **0.0006**, n = 536, HR = 0.98 (0.97 to 0.99)	*P *= **0.0007**, n = 548, HR = 1.027 (1.012 to 1.04)

Data in bold are significant (*P *< 0.05). Data in parentheses are the 95% confidence intervals. HR, hazard ratio.

**Figure 5:**
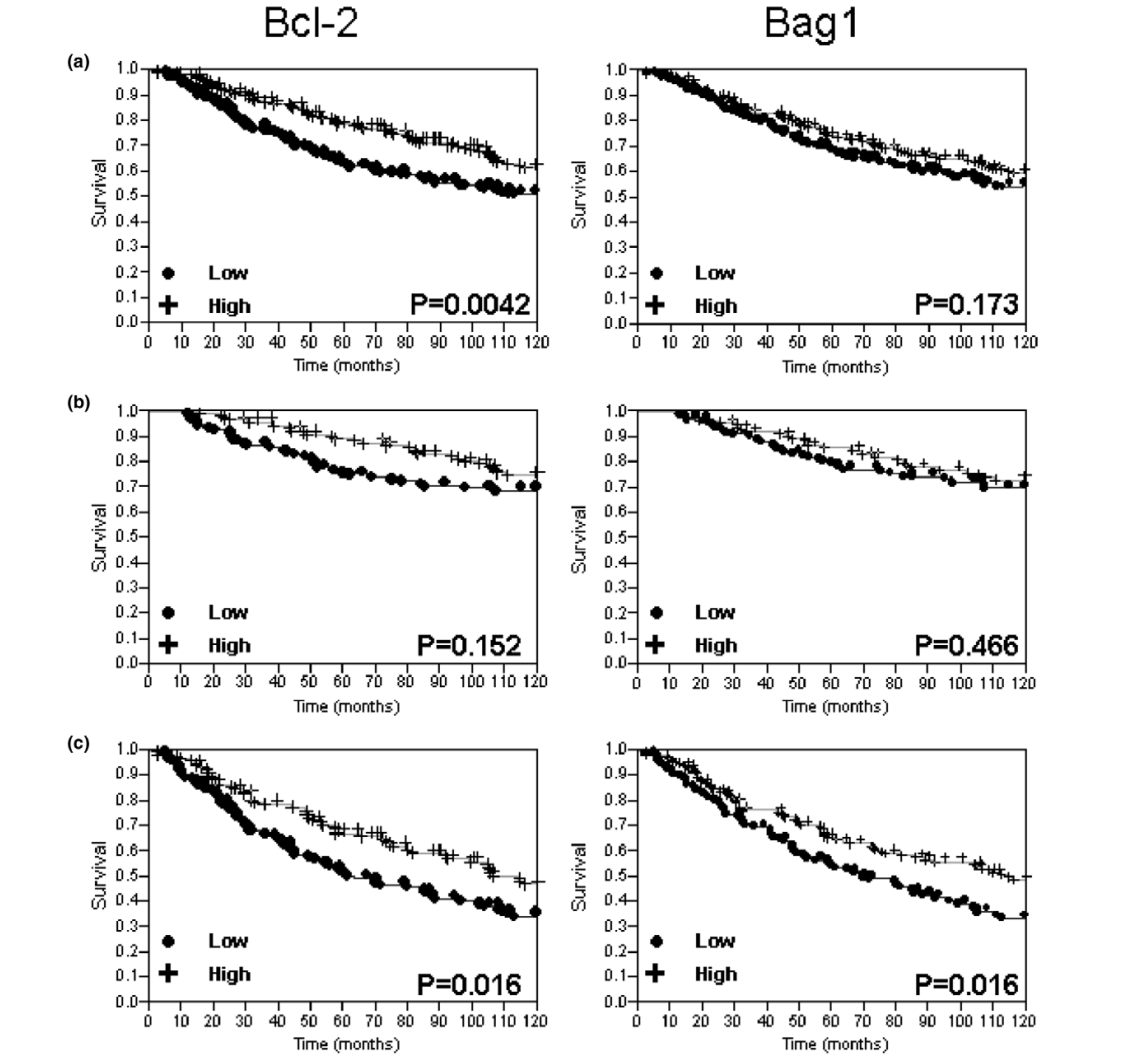
**Kaplan–Meier survival curves for Bcl-2 and Bcl-2 antanogene-1**. Kaplan–Meier survival curves for Bcl-2 and Bcl-2 antanogene-1 (Bag-1) automated quantitative analysis scores dichotomized by the median score for **(a) **the entire cohort of patients, **(b) **node-negative patients, and **(c) **node-positive patients.

Continuous AQUA scores were then dichotomized arbitrarily by the median score, reflecting the use of routine statistical divisions in the absence of an underlying justification for division of expression levels. Kaplan–Meier survival curves were generated for the total Bag-1 score and Bcl-2, as shown in Figure 5. The log-rank analysis revealed a statistically significant association with survival in the node-positive subset for Bcl-2 and Bag-1 (*P *= 0.016 for both markers), and for Bcl-2 in the entire cohort (*P *= 0.0042).

Using the Cox proportional hazards model, we performed multivariable analyses to assess the independent prognostic value of Bag-1 and Bcl-2 expression. Neither of these markers retained their independent prognostic value in this model, and the only markers to be independent predictors of survival were Her2/neu, tumor size and nodal status.

To assess the association between Bag-1 and Bcl-2 expression and other commonly used clinical and pathological parameters, we performed analyses of variance. No associations were found between expression of Bag-1 or Bcl-2 and nodal status, tumor size, age or nuclear grade.

For patients who had AQUA scores for all five biomarkers, we assessed the associations between biomarkers by Spearman's rho test, as demonstrated in Table 2. Bag-1 and Bcl-2 were strongly correlated with the ER, and with the PR but to a lesser degree.

**Table 2:** Spearman's rho associations between expression of Her2/neu, estrogen receptor, progesterone receptor Bcl-2 antanogene-1 (Bag-1) and Bcl-2

	Estrogen receptor	Progesterone receptor	Bag-1	Bcl-2
Her2/neu	-0.0093, *P *= 0.82 (-0.0897 to 0.0712)	-0.066, *P *= 0.93 (-0.146 to 0.0149)	0.0617, *P *= 0.1430 (-0.0209 to 0.1436)	-0.0379, *P *= 0.3885 (-0.1234 to 0.0482)
Estrogen receptor		**0.4942, *P *< 0.0001 (0.4304 to 0.5531)**	**0.4150, *P *< 0.0001 (0.3438 to 0.4814)**	**0.5459, *P *< 0.0001 (0.4824 to 0.6036)**
Progesterone receptor			**0.2260, *P *< 0.0001 (0.1456 to 0.3035)**	**0.3661, *P *< 0.0001 (0.2889 to 0.4385)**
Bag-1				**0.3050, *P *< 0.0001 (0.2228 to 0.3830)**

Data in bold are significant (*P *< 0.05). Data in parentheses represent the 95% confidence intervals.

Figure 1 shows a principal component biplot, in which both patient profiles and biomarker profiles are projected onto the leading principal components. As can be seen, the samples are distributed in a tree-like branch structure. The samples on the branch in the positive direction of the first principal component are associated with high Her2/neu expression levels and low to medium levels of ER, PR, Bag-1 and Bcl-2. Similarly, the samples in the upper left branch have elevated PR expression levels, low to medium ER, Bcl-2 and Bag-1 expression levels, and low Her2/neu expression levels. The samples in the lower left branch have elevated ER, Bcl-2 and Bag-1 levels, low to medium PR levels, and low Her2/neu levels. The bulk of the samples are localized next to the origin, and therefore their inner products with any of the five biomarker vectors are not high – thus indicating that these patients have median or below median expression values across all five biomarkers. Censored patients with less than 10-year follow-up time were omitted from the biplot. The figure further demonstrates the strong association between ER, Bag-1 and Bcl-2.

## Discussion

In the present work we assessed expression of Bcl-2 and Bag-1 in primary breast cancer specimens. Consistent with published reports, we demonstrated that high Bcl-2 expression is associated with improved survival and ER-positive and PR-positive tumors. To the best of our knowledge, this is the first large cohort study assessing Bag-1 expression and its association with Bcl-2, ER, PR and Her2/neu, using continuous output scores, rather than arbitrary pathologist-based divisions of scores into high/low or strong/weak. We demonstrated an association between high Bag-1 expression and survival in the node-positive patients, and found that Bag-1 tends to be coexpressed with Bcl-2, ER and PR. Of the markers studied, the strongest association in expression was found between Bcl-2 and ER. On multivariate analysis, neither Bcl-2 nor Bag-1 retained their independent prognostic value – probably due to the strong association with ER and PR expression. One prior report in the literature used a cohort of 920 patients, and showed that Bcl-2 was an independent prognostic marker. The smaller size of the present cohort might account for our inability to reproduce this result [6].

Staging of primary breast cancer is performed to determine prognosis and to select adjuvant therapies, which decrease the risk of relapse and death. Standard staging criteria are beneficial for discriminating between patients, but within each stage group there is variability in outcome and in biological profiles of tumors. Molecular markers such as Bcl-2 and Bag-1 expression could supplement our standard staging information. Adjuvant chemotherapy decreases the risk of death by approximately 50% in node-positive patients, and the current standard of care in the United States includes relatively aggressive regimens using multiple chemotherapy agents, usually given in a dose-dense fashion [2,71]. The benefit from aggressive chemotherapy regimens for node-positive breast cancer is much lower for patients with hormone receptor-positive tumors than for those with ER/PR-negative tumors [71]. Markers of improved prognosis (such as Bag-1 and Bcl-2) in this group of patients could therefore enable us to determine which patients are cured without adjuvant chemotherapy, thus avoiding the toxicity and cost associated with chemotherapy.

The biological basis for the association between high Bcl-2 and high Bag-1 expression and improved survival has yet to be determined. Given that both of these proteins are key anti-apoptotic mediators that are part of the mitochondrial (indirect) pathway [72], one would expect their expression would be associated with decreased survival, rather than with increased survival. One plausible explanation is that poorly differentiated tumors depend on other prosurvival pathways, and decreased Bcl-2 and Bag-1 expression is merely a marker of aggressive tumor behavior rather than mechanistically associated with aggressive biology. Breast cancer studies in the literature have consistently demonstrated an association between high Bcl-2 expression and improved survival [4-24], and similar findings have been demonstrated in other diseases [73].

Another possible explanation for our findings is that in good-prognosis breast cancer tumors, Bcl-2 and Bag-1 have other dominant roles that are not related to their anti-apoptotic functions. For example, studies have shown that high levels of Bcl-2 actually inhibit cell growth [74,75]. Most of the literature, however, supports an anti-apoptotic role of Bcl-2 and Bag-1 in early-stage breast cancer, which appears to be related to the transcriptional function of ER. Nuclear Bag-1 stimulates the activity of the α and β subunits of the ER [46], and this might be the basis for coexpression of ER and Bag-1 in human tumors. The PR is a transcriptional target of the ER, and would thus similarly be expected to have coexpression with Bag-1. ER transcriptional activity results in Bcl-2 upregulation in breast cancer, and both ER and Bcl-2 are associated with chemotherapy resistance in breast cancer [76,77]. The strong association between ER, PR, Bcl-2 and Bag-1 in our study suggests that co-targeting these molecules in hormone receptor-positive breast cancer might provide greater benefit than chemotherapy, or might be a beneficial strategy for sensitizing these tumors to chemotherapy – including in the node-positive subset of patients.

Drugs that target Bcl-2 are already in clinical trials. Antisense to Bcl-2 has activity in breast cancer in preclinical models as a single agent and also sensitizes high Bcl-2-expressing cells to a range of chemotherapeutic agents [26]. A randomized clinical trial for metastatic melanoma comparing dacarbazine alone with dacarbazine and Bcl-2 antisense demonstrated an increase in response rates and improved survival in patients with less aggressive disease, but not in patients with more aggressive disease (as assessed by levels of lactate dehydrogenase (LDH) [78]. Unfortunately, expression of Bcl-2 was not assessed in this clinical trial; however, previous studies on metastatic melanoma specimens suggest that patients with more advanced disease have significantly lower levels of Bcl-2 expression [73]. Additional small-molecule inhibitors that target Bcl-2 are currently being studied [79,80]. Attempts at targeting Bag-1 as a sensitizer to chemotherapy are in their infancy [81], and our data suggest that combinations of antihormonal therapy with drugs that target Bcl-2 and/or Bag-1 should also be investigated, particularly in patients with high Bcl-2 or Bag-1 levels.

## Conclusion

In summary, using an automated quantitative method of protein expression analysis to study a large cohort of primary tumors, we have shown that both Bcl-2 and Bag-1 are associated with improved survival in node-positive breast cancer, and that both Bcl-2 and Bag-1 are strongly associated with ER and PR expression. Targeting Bcl-2 and/or Bag-1 in node-positive, hormone receptor-positive breast cancer might improve the therapeutic ratio of adjuvant therapy in this population. Future clinical trials using agents that target Bcl-2 and/or Bag-1 in breast cancer should focus on hormone receptor-positive patients, and all studies should incorporate assessment of target expression in pretreatment tumors to assess the association between target expression and response to therapy.

## Abbreviations

AQUA = automated quantitative analysis; Bag-1 = Bcl-2 antanogene-1; ER = estrogen receptor; PR = progesterone receptor.

## Competing interests

RLC and DLR are founders, stockholders, and consultants of HistoRx, a private corporation to which Yale University has given exclusive rights to produce and distribute software and technologies embedded in AQUA. Yale University retains patent rights for the AQUA technology. The other authors declare that they have no competing interests.

## Authors' contributions

HMK and YK contributed equally to this work. YK and HMK initiated the project, performed all the computational aspects of the projects and drafted the manuscript. YN performed the experiments for BAG-1 and Bcl-2, and assisted in drafting the manuscript. RLC and DLR developed the AQUA technology. JMG and CM performed the AQUA analysis for Her2, ER and PR. All authors read and approved the manuscript.
